# Advancing Global Nursing Policy: AI‐Driven Health Literacy Innovations for Migrant Domestic Workers

**DOI:** 10.1111/inr.70149

**Published:** 2026-01-21

**Authors:** Pak Leng Cheong, Kazumi Kubota

**Affiliations:** ^1^ Education Department Kiang Wu Nursing College of Macau Macau China; ^2^ Research Organization Shimonoseki City University Yamaguchi Japan; ^3^ Department of Healthcare Information Management The University of Tokyo Hospital Tokyo Japan; ^4^ Health and Global Policy Institute Tokyo Japan

**Keywords:** domestic workers, digital health, health equity, health literacy, migrant workers, multilingual communication, nursing informatics, nursing policy

## Abstract

**Aim:**

This perspective proposes evidence‐informed strategies to advance health literacy equity for migrant domestic workers (MDWs) globally, integrating research findings and outlining nursing‐led policy directions for sustainable change.

**Background:**

MDWs face language barriers, precarious employment conditions, social isolation, and systemic discrimination that restrict healthcare access and preventive care. Digital exclusion further limits their ability to navigate health information and services effectively.

**Sources of Evidence:**

The analysis synthesizes evidence from scoping reviews, mixed‐methods and quasi‐experimental intervention studies, chronic disease health literacy surveys, artificial intelligence guidance literature, and qualitative research on MDWs’ technology use and peer health information‐sharing practices.

**Discussion:**

Mobile health applications, culturally tailored social media campaigns, and AI‐powered chatbots can deliver accessible health education when co‐designed with MDWs and supported by community health workers. Critical policy actions include bridging the digital divide, embedding health literacy in orientation programs, guaranteeing paid medical leave, ensuring professional interpretation, and establishing accountability mechanisms.

**Conclusion:**

Digitally supported interventions grounded in culturally responsive approaches can reduce health literacy disparities among MDWs. Sustainable impact requires coordinated cross‐sector collaboration spanning health, labor, education, and technology sectors.

**Implications for Nursing Practice:**

Nurses should lead participatory co‐design of digital health tools with MDWs, adopt universal health literacy precautions, and integrate teach‐back methods and visual aids into routine clinical practice.

**Implications for Nursing Policy:**

Policy frameworks must mandate health literacy standards, establish language access requirements, allocate resources for multilingual materials and professional interpretation services, support digital inclusion initiatives, and integrate MDW health literacy indicators into routine monitoring systems.

## 1 Aim

This article offers a policy innovation perspective for promoting health literacy equity among migrant domestic workers (MDWs) globally. Drawing on a health equity‐oriented scoping review of care transitions for refugees and migrants (Liu et al. 2025), a mixed‐methods investigation of eHealth literacy among MDWs in Hong Kong (Milanti et al. 2024), quasi‐experimental community interventions in Europe (Al‐Adhami et al. 2024), and prevalence of hypertensive health literacy among Myanmar workers in Thailand (Thi and Sornlorm 2023), this article integrates conceptual frameworks including the Health Literacy Skills Framework (Squiers et al. 2012) and a social determinants of health perspective on migrant populations (Ward et al. 2019). It explores how systemic barriers—such as adverse labor conditions and discrimination documented among Indonesian domestic workers in Singapore (Platt et al. 2016) and the complexity of host‐country healthcare systems intersect with individual abilities. The discussion focuses on the dual role of technological innovation and community engagement: evaluating mobile health apps (Fernández‐Gutiérrez et al. 2019), social media initiatives (Whitehead et al. 2023), AI‐driven chatbots with transparency and ethical oversight guidance (Adegboye 2024), and extended health communication sessions (Al‐Adhami et al. 2024). Through this lens, this article aims to illustrate actionable policy directions, such as guaranteed medical leave, embedding health literacy in settlement orientation services, making professional interpretation and multilingual materials mandatory, and establishing digital inclusion initiatives (Yameogo et al. 2023; Kickbusch and Gleicher 2012; Wahidie et al. 2024). Nursing professionals ought to spearhead the co‐design of these interventions, advocating for cross‐sector collaboration across health, labor, education, and technology sectors, and shaping governance frameworks that ensure the right of MDWs to have accessible, understandable, and usable health information and services.

## 2 Background

Health literacy (i.e., the ability to obtain, understand, and act on health information) is fundamental to achieving good health outcomes (Sørensen et al. 2012). According to the International Labour Organization (2021), roughly 75.6 million people work as domestic workers worldwide. Of these, 76.2% are women and 17.2% are international migrants. This heavily feminized, mobile workforce faces health challenges shaped by the intersection of gender, migration status, and precarious employment (Benach et al. 2011).

MDWs commonly encounter language and cultural barriers that complicate communication with healthcare providers (Szczepura 2005). Many live‐in employer households under conditions marked by substandard accommodation, below‐minimum wages, excessive working hours, and limited personal freedom (Human Rights Watch 2014). Social isolation—a frequent consequence of live‐in arrangements—restricts access to peer support and community resources (Vahabi and Wong 2017). Together, these social determinants heighten exposure to infectious disease, amplify psychological distress, and curtail opportunities for preventive care (Castañeda et al. 2015).

Gender adds another layer of complexity. Women MDWs often lack access to reproductive and maternal health information, face cultural taboos around discussing sexual health, and may experience workplace harassment or violence without clear avenues for reporting or redress (Piper 2009). Dependency on employers for housing and legal status can make it especially difficult to seek help (Amnesty International 2013).

Systemic factors compound individual vulnerabilities. Language mismatches and unfamiliarity with host‐country healthcare systems discourage timely care‐seeking (Wahidie et al. 2024). Chronic disease risks, cardiovascular conditions, for example, are elevated when culturally appropriate preventive information is unavailable (Bulto and Hendriks 2025). The COVID‐19 pandemic underscored these dynamics: MDWs faced higher infection rates alongside barriers to accurate, timely guidance (Liem et al. 2020).

As health services move online, digital exclusion has emerged as a critical concern. Many MDWs lack affordable internet access, suitable devices, or the confidence to use digital tools effectively (Whitehead et al. 2023; Yameogo et al. 2023). At the same time, research shows that MDWs actively use smartphones and messaging apps to share health knowledge and compensate for gaps in formal support (Platt et al. 2016). Recognizing this duality, the present article frames MDW health literacy as a matter requiring attention to both individual capabilities and structural reform.

### 2.1 Sources of Evidence

A growing body of peer‐reviewed research supports this policy perspective. A health equity‐oriented scoping review examined care transition interventions for refugee, immigrant, and migrant populations, identifying that programs featuring health navigation or public health education, tailored to language, culture, and educational level, yielded the most promising outcomes (Liu et al. 2025). In Hong Kong, a mixed‐methods study identified a gap in digital health literacy among Filipino MDWs during the COVID‐19 pandemic, despite their frequent use of social media platforms for health information (Milanti et al. 2024). A quasi‐experimental evaluation of the “e_SaludAble” mobile health apps in Spain demonstrated significant improvements in functional health literacy among immigrant users following tailored training and app utilization (Fernández‐Gutiérrez et al. 2019). Parallel research in Sweden showed that extended health communication sessions integrated into refugee settlement programs significantly improved health literacy scores, suggesting this approach is adaptive to the orientation processes for MDWs (Al‐Adhami et al. 2024).

Furthermore, a survey of primary care providers in Hessen, Germany, not only highlighted reliance on multilingual materials, bilingual staff, and digital translation tools to support migrant patients but also revealed gaps in consultation time and standardized workflows for health literacy (Wahidie et al. 2024). Additional evidence from the European Union—including a systematic review and a primary‐care study—shows that migrants exhibit lower health literacy levels than native‐born populations, with downstream impacts on care utilization and outcomes (Ward et al. 2019; Medina et al. 2022). Qualitative analyses demonstrate how Indonesian domestic workers in Singapore navigate migration experiences by leveraging information and communication technologies—cell phones, messaging apps, and online forums—to compensate for formal support deficits and share health knowledge within peer networks (Platt et al. 2016). In northeastern Thailand, high prevalence of limited hypertensive health literacy among Myanmar migrant workers was linked to education level and duration of residence, underscoring the need for targeted literacy support in chronic disease management (Thi and Sornlorm 2023). Finally, a theoretical discussion on artificial intelligence's ramifications for health information literacy urges the development of guidance and standards for AI‐generated content, as healthcare professionals increasingly encounter AI‐driven tools (Adegboye 2024).

Table 1 summarizes key evidence from studies involving MDWs as well as broader migrant and refugee populations whose structural conditions (e.g., language barriers, precarious work, limited access to services) closely resemble those of MDWs.

**TABLE 1:** Summary of key evidence informing policy recommendations relevant to MDW health literacy.

Author(s), year	Setting	Population	Study design	Main findings	Nursing policy implications
Liu et al. (2025)	Global	Refugees, immigrants, migrants	Scoping review	Programs combining health navigation with culturally tailored education yielded the strongest outcomes	Although not MDW‑specific, design interventions around language, culture, and education level; embed navigation support in care pathways that can be adapted for MDWs.
Milanti et al. (2024)	Hong Kong	Filipino MDWs	Mixed methods	Despite frequent social media use, eHealth literacy remained low during COVID‐19	Digital access alone is insufficient; targeted literacy training and skills‐building are essential
Fernández‐Gutiérrez et al. (2019)	Spain	Immigrants	Quasi‐experimental	“e_SaludAble” mHealth app combined with training significantly improved functional health literacy	Participatory app design and structured hands‐on training enhance intervention effectiveness
Al‐Adhami et al. (2024)	Sweden	Newly settled refugees	Quasi‐experimental	Extended health communication sessions raised literacy scores by over 20%	Integrate structured health education into settlement and orientation programs
Thi and Sornlorm (2023)	Northeastern Thailand	Myanmar migrant workers	Cross‐sectional survey	High prevalence of limited hypertensive health literacy, associated with education level and residence duration	Chronic disease literacy requires targeted, sustained support tailored to worker characteristics
Wahidie et al. (2024)	Hessen, Germany	Primary care providers	Online survey	Providers rely on multilingual materials and translation tools but lack adequate time and standardized protocols	Institutionalize health literacy workflows in primary care; allocate sufficient consultation time
Platt et al. (2016)	Singapore	Indonesian MDWs	Qualitative	MDWs actively use ICT (phones, messaging apps, forums) to share health information and compensate for formal support gaps	Leverage existing peer networks and technology practices when designing formal interventions
Adegboye (2024)	Global	Healthcare professionals	Theoretical discussion	AI‐generated health content requires professional guidance, accuracy standards, and ethical oversight	Develop AI governance frameworks addressing accuracy, bias, transparency, and accountability

*Note*: Some studies in this table focus on refugees or migrants in general rather than MDWs specifically, but they are included because their findings are transferable to MDWs given the similar social and structural determinants of health.

Looking across these sources, four themes stand out. First, interventions tailored to language, culture, and educational background consistently outperform generic approaches (Liu et al. 2025; Fernández‐Gutiérrez et al. 2019). Second, digital tools show genuine promise but work best when paired with human support—whether from community health workers, peers, or clinicians (Al‐Adhami et al. 2024; Whitehead et al. 2023). Third, structural conditions—labor arrangements, employer relationships, healthcare system design—shape literacy outcomes as powerfully as individual‐level factors (Platt et al. 2016; Wahidie et al. 2024). Fourth, informal peer networks already serve as important channels for health information among MDWs, representing an underutilized resource for formal programs (Platt et al. 2016).

At the same time, the evidence base has clear limitations that must inform policy development. Most intervention studies used quasi‐experimental designs without randomization, constraining causal claims (Fernández‐Gutiérrez et al. 2019; Al‐Adhami et al. 2024). Follow‐up periods were typically short—often under six months—leaving questions about long‐term sustainability unanswered (Nutbeam et al. 2018). Sample sizes tended to be modest, and the geographic spread was narrow: Hong Kong, Spain, Sweden, Thailand, Germany, and Singapore feature prominently, but many major migration corridors remain unstudied. Few investigations measured downstream health outcomes such as disease control or service utilization; most focused on intermediate literacy metrics (Berkman et al. 2011). Finally, given how rapidly digital technology evolves, findings on specific apps or platforms may date quickly, reinforcing the need for ongoing evaluation of emerging tools, including AI‐driven applications (Adegboye 2024). These gaps highlight the importance of investing in rigorous, longitudinal, multi‐site research to guide evidence‐based policy for MDW health literacy.

## 3 Discussion

### 3.1 Health System Responsiveness and Cross‐Sector Collaboration

Evidence underscores that health systems must move beyond ad hoc translation services to deeply embed health literacy strategies within service design. Institutionalizing multilingual portals, professional interpretation, and easy‐read materials must be accompanied by clinician training in teach‐back methods and plain‐language communication. Public health agencies should partner with migrant support NGOs, labor organizations, and community groups to deliver health information within workers’ living and workplace contexts. For instance, collaboration between nursing associations and employers to schedule regular health workshops or worker gatherings at community centers can promote trust and uptake. Policy frameworks should align labor regulations with public health goals by providing for guaranteed leave to healthcare access and requiring employers to facilitate information sessions in the native languages of workers.

### 3.2 Effectiveness of Digital and Community‐Based Interventions

Digital health holds considerable promise for bridging health literacy gaps, provided the underlying digital divide is addressed through subsidized access and skills training (Latulippe et al. 2017).

Mobile health applications developed with MDW input can deliver tailored education, appointment reminders, medication support, and symptom guidance. The “e_SaludAble” evaluation in Spain offers strong evidence that culturally adapted apps, combined with structured training, produce measurable improvements in functional health literacy among immigrant users (Fernández‐Gutiérrez et al. 2019). Germany's Zanzu platform—offering sexual and reproductive health information in 13 languages with visual explanations—demonstrates how multilingual digital resources can be designed for diverse migrant audiences (BZgA 2023). Singapore's HealthHub provides multilingual access to personal health records, though uptake among MDWs remains limited by awareness gaps and digital literacy barriers (Ministry of Health Singapore 2022).

Social media campaigns on platforms popular with MDW communities—Facebook, WhatsApp, and regional apps—can spread culturally resonant health messages and counter misinformation (Betsch et al. 2020). Research during COVID‐19 showed that peer‐shared content through trusted networks achieved higher engagement among migrant groups than official institutional messaging (Guadagno 2020).

AI‐powered chatbots offer the attraction of round‐the‐clock, multilingual support, but they introduce distinct ethical challenges that demand careful attention (Miner et al. 2020). Algorithmic bias is a primary concern: systems trained mainly on English‐language medical literature and data from high‐income settings may give less accurate or culturally inappropriate responses to MDWs from different backgrounds (Obermeyer et al. 2019). Transparency matters too—users should know they are interacting with AI, not a human, and should understand the limits of machine‐generated advice (Vayena et al. 2018). Privacy protections must be robust, especially given MDWs’ precarious immigration and employment status; health data could potentially be accessed by employers or authorities, with serious consequences (Torous and Roberts 2017). Data governance frameworks need to specify collection, storage, retention, and deletion rights.

Hallucinations—cases where AI confidently produces factually wrong information—pose particular dangers in health contexts. A chatbot that invents medication dosages or mischaracterizes symptoms could lead users to delay necessary care or take harmful actions. Mitigation strategies include mandating human review of AI health recommendations, conducting regular accuracy audits against clinical guidelines, and building in clear pathways for users to reach human providers (World Health Organization 2023). The World Health Organization (2023) emphasizes that AI in health should augment, not replace, human judgment—a principle especially important for vulnerable populations.

None of this diminishes the value of human support. Community health workers and peer navigators remain essential for introducing digital tools, demonstrating their use, building confidence, and providing encouragement (Tran et al. 2004). Hybrid approaches—combining apps or chatbots with drop‐in clinics for device training, or community center workshops—tend to achieve the best engagement (Latulippe et al. 2017). Sweden's extended health communication model shows that weaving digital literacy into broader settlement education can yield durable gains (Al‐Adhami et al. 2024). Employer engagement offers another avenue: partnerships between nursing bodies, labor organizations, and employers to host periodic health sessions at community venues can extend reach, though careful attention to power dynamics and voluntary participation is essential (Piper and Withers 2018).

### 3.3 Multilingual Tools and Translation

True multilingual communication must go beyond simple translation. Health materials should be co‐developed with MDWs to ensure cultural relevance and clarity. Visual aids—infographics, videos with captions, and interactive voice response systems—can overcome literacy barriers. Technology can support on‐demand translation kiosks in clinics, but critical medical advice should be validated by bilingual healthcare professionals. Policy should require all public health campaigns and emergency alerts to be disseminated in major MDW languages and dialects, with accessible formats for low‐literacy audiences.

### 3.4 Persistent Gaps: Evaluation, Scalability, and Cost‐Effectiveness

Despite promising pilots, rigorous long‐term evaluations remain scarce. Most studies report short‐term literacy gains, but whether these translate into sustained behavioral change and improved health outcomes is largely unknown (Nutbeam et al. 2018). Longitudinal follow‐up extending beyond 12 months is essential to determine intervention durability.

A robust evaluation framework for MDW health literacy programs should address three dimensions. First, outcome evaluations must go beyond literacy scores to capture downstream effects—healthcare utilization patterns, preventive service uptake, chronic disease indicators, and self‐reported health status (Berkman et al. 2011). Second, process evaluations should examine implementation fidelity, user engagement, and the contextual factors that shape uptake across different MDW populations (Proctor et al. 2011). Third, equity‐focused analyses must disaggregate findings by language, nationality, education, and employment conditions to detect differential impacts and avoid one‐size‐fits‐all conclusions (Lorenc et al. 2013).

For AI‐driven interventions, additional evaluation considerations apply, particularly systematic monitoring for hallucinations—confident but factually wrong outputs—and other errors. Evaluation protocols should therefore include regular accuracy audits benchmarking AI outputs against validated clinical guidelines, systematic tracking of error patterns, and user comprehension checks to see whether MDWs can distinguish reliable from unreliable information (World Health Organization 2023). Bias assessments are equally important: AI tools trained primarily on English‐language data and Western healthcare contexts may perform poorly for MDWs from different linguistic and cultural backgrounds (Obermeyer et al. 2019). Privacy impact assessments should also be standard, given that MDWs’ vulnerable immigration and employment status makes health data particularly sensitive (Vayena et al. 2018).

Scalability requires standardized yet adaptable toolkits and training modules (Damschroder et al. 2009). International collaboration—through shared repositories of validated multilingual content and best practices—can accelerate progress. National accreditation bodies might incentivize health literacy planning by incorporating relevant metrics into quality certification schemes (Brach et al. 2012). Above all, policymakers should commit funding to extended follow‐up studies and comparative cost‐effectiveness analyses across digital, community‐based, and hybrid models.

### 3.5 Integrated Policy Action Across Sectors

A whole‐of‐government approach is essential. Health, labor, education, and technology ministries must jointly develop national health literacy strategies encompassing MDWs. Education systems can incorporate health modules into language classes. Labor inspectors can assess compliance with health rights during workplace audits. Telecommunication providers can partner to offer subsidized data plans for health apps. Global entities such as thWHO and IOM should include MDW health literacy in migration compacts and Sustainable Development Goal monitoring, ensuring no worker is left behind in the digital transformation of healthcare.

### 3.6 Implications for Nursing Practice and Policy

#### 3.6.1 Implications for Nursing Practice

Nurses are well placed to work directly with MDWs and community health workers in co‐designing digital health tools that genuinely reflect user needs (Greenhalgh et al. 2017). Co‐design is more than consultation; it means involving MDWs as partners throughout the development process. Practical approaches include community‐based participatory research workshops held on MDWs’ rest days at accessible venues (Israel et al. 2010), focus groups organized by language and country of origin to capture diverse perspectives, iterative prototype testing with real users to refine usability, and standing advisory panels that keep MDW voices central even after initial development ends. Evidence from the “e_SaludAble” project confirms that participatory methods improve both usability and health outcomes (Fernández‐Gutiérrez et al. 2019).

Nursing informatics specialists bring a distinctive skill set to AI‐enabled intervention development. Trained at the intersection of clinical care and information systems, they can ensure that chatbots and decision‐support tools use plain language consistent with health literacy principles, incorporate appropriate clinical safeguards, and maintain transparent privacy controls (Booth et al. 2021). In practice, this might involve establishing validation routines that check AI outputs against evidence‐based guidelines, creating escalation pathways so complex questions reach human clinicians, and training MDW users to critically assess machine‐generated advice (World Health Organization 2023).

Rather than relying solely on individual assessments, nursing practice should embrace a universal health literacy precautions approach (Brach, 2024). This means assuming that every MDW will benefit from clear, plain‐language communication—not just those identified as having limited literacy. Such an approach reflects the reality that even highly literate individuals may struggle when facing an unfamiliar diagnosis, navigating a foreign healthcare system, or experiencing significant stress (Koh et al. 2012). By consistently using teach‐back methods, visual aids, and simplified explanations for all patients, nurses can reduce stigma and ensure that no one falls through the cracks (Brach, 2024). The Health Literacy Skills Framework (Squiers et al. 2012) remains useful for understanding how individuals process health information, but it should inform communication strategies universally applied rather than trigger selective interventions.

#### 3.6.2 Implications for Nursing Policy

Sustainable progress requires coordinated policy action at multiple levels (Kickbusch and Gleicher 2012). National health ministries should set overarching frameworks—mandating health literacy standards, establishing language access requirements, and allocating resources for multilingual materials and interpreter services (Brach et al. 2012). Regional and local health authorities can then adapt these frameworks to the specific MDW populations and healthcare infrastructures in their areas. Clear delineation of responsibilities prevents duplication and ensures accountability (World Health Organization 2016).

Several national models offer useful reference points. Australia's National Health Literacy Strategy embeds literacy considerations across health system planning, workforce training, and consumer engagement, providing a template that could be extended to MDW‐inclusive policies (Australian Commission on Safety and Quality in Health Care, 2014). In the Netherlands, the Pharos program integrates migrant health indicators into primary care quality metrics, giving providers concrete incentives to attend to culturally appropriate communication (Pharos 2021). Thailand's migrant health insurance scheme demonstrates how coverage can be extended to documented migrant workers, improving access—though domestic workers often remain excluded, underscoring the need for occupation‐specific attention (Suphanchaimat et al. 2019).

Digital inclusion deserves targeted policy support. National strategies might include subsidized smartphone and data programs for domestic workers, public–private partnerships with telecom providers for reduced‐cost connectivity, and community‐based device donation or refurbishment initiatives (Robinson et al. 2015). Such measures help ensure that digital health innovations reach those who stand to benefit most.

Monitoring and accountability mechanisms are essential for sustained impact. Health ministries should define MDW health literacy indicators and integrate them into routine health information systems, with data disaggregated by nationality, language, and employment type to reveal disparities. Periodic policy audits can assess whether mandated services—interpretation, multilingual materials, provider training—are actually being delivered (Rechel et al. 2013). Independent evaluations, whether by academic partners or oversight bodies, add external verification and can inform course corrections.

Finally, cross‐sector governance is indispensable (Kickbusch and Gleicher 2012). Labor regulations should guarantee paid medical leave so MDWs can access care without risking their jobs. Immigration policies should, where feasible, delink healthcare entitlements from employer sponsorship (Abubakar et al. 2018). Transition‐in‐care frameworks (Liu et al. 2025) highlight the importance of seamless navigation between employer‐mediated living situations and public health services. By embedding these principles in labor, health, and immigration law, governments can create the structural conditions within which AI‐enhanced, nurse‐led health literacy initiatives can genuinely flourish.

## 4 Conclusion

Enhancing health literacy among MDWs is both a public health imperative and a matter of social justice. Equipping MDWs with the skills and resources to understand and act on health information empowers individuals and strengthens community resilience. Digital innovations—when co‐designed with MDWs, supported by community health workers, and embedded in inclusive policies—can bridge long‐standing gaps. Several factors position nurses to take the lead in this work. Nurses represent the largest professional group in healthcare and typically have the most frequent and sustained contact with patients across settings—from community clinics to hospital wards (World Health Organization 2020). This proximity creates ongoing opportunities to identify literacy challenges and provide timely education. Nursing education emphasizes holistic, people‐centered care that accounts for social context, cultural background, and family dynamics—competencies highlighted in global nursing policy frameworks and closely aligned with the needs of MDWs (World Health Organization 2020). Nurses also bring well‐established skills in health teaching, patient communication, and care coordination that translate directly to designing and delivering literacy interventions (Nutbeam and Lloyd 2021). Historically, the profession has championed advocacy for marginalized groups, making social justice a natural extension of nursing practice (Falk‐Rafael 2005). Finally, nursing informatics has matured into a recognized specialty that bridges clinical expertise with health information technology, equipping nurses to guide the responsible development and deployment of AI‐enabled tools while keeping patient welfare at the center (Booth et al. 2021). Nursing leadership is crucial in advocating for policy change, co‐creating interventions, and training the workforce in digital and cultural competence. Through coordinated action and sustained evaluation across sectors, the vision of health for all can extend to those who care for others but remain on the margins of health systems.

## Author Contributions

Study concept and design: PLC and KK. Acquisition of data: KK and PLC. Analysis and interpretation of data: PLC and KK. Manuscript writing: KK. Critical revision of the manuscript for important intellectual content: PLC.

## Funding

The authors have nothing to report.

## Ethics Statement

Ethics approval and informed consent were not required because this manuscript is a non‐empirical analysis based on publicly available literature and does not involve the collection or analysis of identifiable individual‐level data.

## Use of Artificial Intelligence

A generative artificial intelligence tool (ChatGPT, OpenAI) was used to support language editing, organization of arguments, and checking of consistency. The authors carefully reviewed, edited, and take full responsibility for all content.

## Conflicts of Interest

No conflict of interest has been declared by the author(s).

## Data Availability

No new datasets were generated or analyzed in the preparation of this manuscript.
